# Capturing molecular structural dynamics by 100 ps time-resolved X-ray absorption spectroscopy

**DOI:** 10.1107/S0909049508034596

**Published:** 2008-11-21

**Authors:** Tokushi Sato, Shunsuke Nozawa, Kohei Ichiyanagi, Ayana Tomita, Matthieu Chollet, Hirohiko Ichikawa, Hiroshi Fujii, Shin-ichi Adachi, Shin-ya Koshihara

**Affiliations:** a Department of Materials Science, Tokyo Institute of Technology, 2-12-1-H61 Ohokayama, Meguro-ku, Tokyo 152-8551, Japan; b Non-Equilibrium Dynamics Project, ERATO, Japan Science and Technology Agency, 1-1 Oho, Tsukuba, Ibaraki 305-0801, Japan; c Institute for Molecular Science and Okazaki Institute for Integrative Bioscience, Myodaiji, Okazaki 444-8787, Japan; d High Energy Accelerator Research Organization, 1-1 Oho, Tsukuba, Ibaraki 305-0801, Japan; e Frontier Research Center, Tokyo Institute of Technology, 2-12-1 Ohokayama, Meguro-ku, Tokyo 152-8551, Japan

**Keywords:** time-resolved X-ray absorption, spin crossover, tris(1,10-phenanthrorine)iron(II)

## Abstract

An experimental set-up for time-resolved X-ray absorption spectroscopy with 100 ps time resolution at beamline NW14A at the Photon Factory Advanced Ring is presented. The X-ray positional active feedback to crystals in a monochromator combined with a figure-of-merit scan of the laser beam position has been utilized as an essential tool to stabilize the spatial overlap of the X-ray and laser beams at the sample position. As a typical example, a time-resolved XAFS measurement of a photo-induced spin crossover reaction of the tris(1,10-phenanthrorine)iron(II) complex in water is presented.

## Abstract 2

An experimental set-up for time-resolved X-ray absorption spectroscopy with 100 ps time resolution at beamline NW14A at the Photon Factory Advanced Ring is presented.

## 1. Introduction

Ultrafast time-resolved X-ray measurements such as diffraction, scattering, absorption and imaging using synchrotron radiation sources are becoming general and powerful tools for exploring structural dynamics in the fields of materials and biological sciences (Šrajer *et al.*, 1996 ▶; Collet *et al.*, 2003 ▶; Schotte *et al.*, 2003 ▶; Cavalleri *et al.*, 2005 ▶; Ihee *et al.*, 2005 ▶; Jeong *et al.*, 2005 ▶; Gawelda *et al.*, 2007 ▶; Ichiyanagi *et al.*, 2007 ▶; Kong *et al.*, 2008 ▶; Wang *et al.*, 2008 ▶). Among these time-resolved X-ray techniques, time-resolved X-ray absorption spectroscopy (TR-XAS) provides dynamic information on the electronic state and molecular structure of non-crystalline samples with temporal resolution of the pulse width of X-rays (Saes *et al.*, 2003 ▶, 2004 ▶; Cavalleri *et al.*, 2005 ▶; Khalil *et al.*, 2006 ▶; Gawelda *et al.*, 2007 ▶). TR-XAS can reveal the dynamics of the oxidation and valence states *via* XANES and the local molecular structure *via* EXAFS with sub-angstrom spatial resolution, which compliments diffraction and scattering techniques (Ihee *et al.*, 2005 ▶; Kong *et al.*, 2008 ▶). Several groups have been developing TR-XAS using synchrotron facilities, and this method has been successfully applied to probe the dynamics of the spin crossover system (Khalil *et al.*, 2006 ▶; Gawelda *et al.*, 2007 ▶) and the photodissociation of ligands in NiTPP (Chen *et al.*, 2001 ▶).

For successful TR-XAS measurement in the pump–probe mode, precise spatial and temporal overlap between the X-ray and laser beam is essential. However, maximizing the incident X-ray beam intensity causes difficulties in keeping the spatial overlap during the experiment. The intensity of the incident X-ray beam is usually maximized to obtain as strong a time-dependent signal as possible, whereas most of the incident photons are wasted by X-ray chopper or electronic gating in order to reduce the repetition rate of the X-ray. The maximized X-ray beam gives a large heat load to X-ray optics, mainly to the monochromator or mirror, which destabilizes the X-ray beam position. In order to maintain the spatial overlap during data collection, the X-ray beam position must be stabilized by active positional feedback. At the same time, the spatial overlap must be reproducibly tuned during the long data collection period to avoid the mismatch of the X-ray and laser positions owing to positional drift of the two beams. The most effective check is the laser positional scan using the photo-induced X-ray signal itself as a figure of merit of the spatial overlap. Here we report the development of a TR-XAS system equipped with stable alignment of the X-ray and laser beam positions at the beamline NW14A, Photon Factory Advanced Ring, and present an example of TR-XAS measurements using these systems.

## 2. Experimental details

### 2.1. X-ray and laser specifications

The 6.5 GeV Photon Factory Advanced Ring (PF-AR) is a full-time single-bunch synchrotron radiation source especially suited for time-resolved X-ray studies using pulsed X-rays because time-resolved experiments primarily require a relatively sparse bunch-filling mode. Electrons with a ring current of 60 mA (75.5 nC per bunch) are stored in a single bucket with a lifetime of ∼20 h. The frequency of the RF cavities and harmonic number of the PF-AR are 508.58 MHz and 640, respectively, corresponding to a revolution frequency of 794 kHz. As the PF-AR is operated in the single-bunch mode, the X-ray pulses are delivered at a frequency of 794 kHz with a pulse duration of 60 ps (r.m.s.). The PF-AR is operational for ∼5000 h annually, which is a major advantage in terms of time-resolved beam time over other synchrotron facilities.

**Figure 1:**
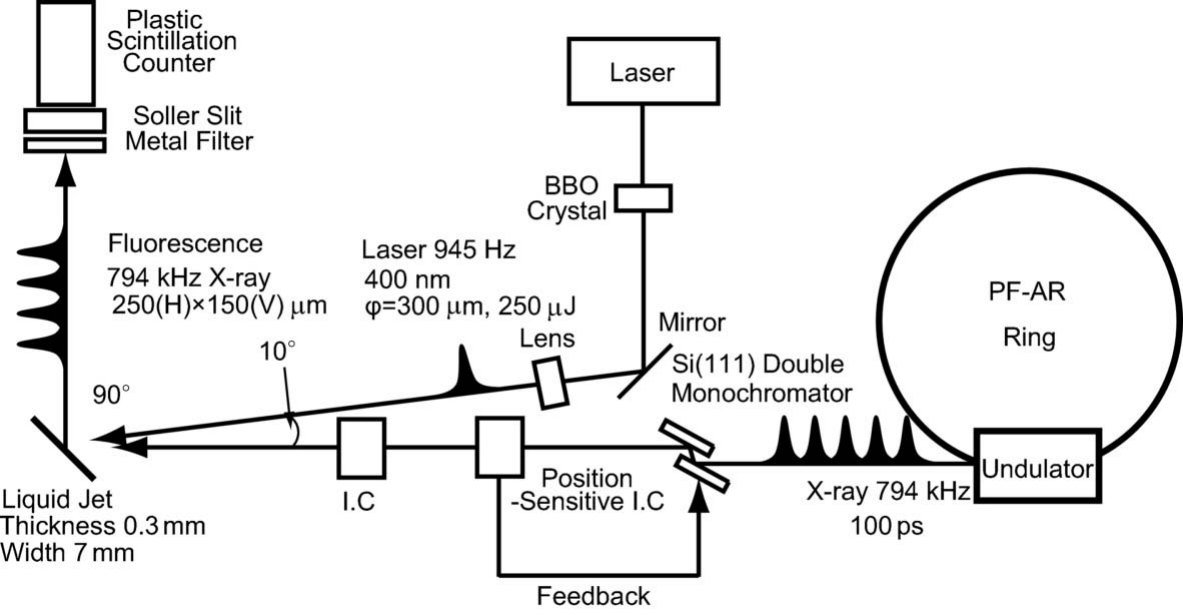
Layout of the time-resolved XAFS experiment at the NW14A beamline at the PF-AR.

In this TR-XAS study, all measurements were performed using the fluorescence XAFS method on the undulator beamline NW14A at the PF-AR, as illustrated in Fig. 1 ▶. Details of the beamline have been reported previously (Nozawa *et al.*, 2007 ▶). The X-ray pulses at 794 kHz were monochromized by a Si(111) monochromator and used for the probe. The energy resolution was Δ*E*/*E* = 1.6 × 10^−4^ at 7 keV, which is estimated from the rocking curve of the Si(111) reflection. The higher harmonics of the reflection were rejected by the double flat mirrors coated with rhodium with a thickness of 100 nm. The X-rays were focused to 250 (H) µm × 150 (V) µm at the sample position by the Rh-coated bent-cylindrical mirror. The intensity of the incident X-ray was detected by ionization chambers.

A 945 Hz regenerative amplified Ti:sapphire laser was used for the pump source. The timing between the X-ray and laser is defined on the basis of the RF master clock that drives the electron bunch in the storage ring. In order to achieve precise synchronization between the laser and X-ray, we used the CANDOX delay-timing system (Ohshima *et al.*, 2007 ▶), which provides an external trigger signal within a 3 ps jitter. This system is composed of frequency dividers, IQ modulator phase shifters and digital counters. The external trigger of the sine wave (84.76 MHz = 508.58 MHz/6) for the mode-locked laser and pulse signal (945 Hz = 508.58 MHz/537600) was provided by the frequency divider. The timing of the laser pulse can be changed by shifting the amount of the IQ modulator phase up to 2 ns (1/508.58 MHz). Delay timing of over 2 ns can be set by the digital counter combined with the IQ modulator up to about 1 ms (1/945 Hz). The laser was converted to 400 nm by a BBO crystal and stretched to a duration of up to 2 ps and focused using a lens with ϕ = 300 µm at the sample position. The angle between the X-ray and the laser was almost parallel (∼10°).

### 2.2. Sample environments, detector and data acquisition

We used an open jet and circulation pump system because liquid samples can be damaged by laser excitation (Saes *et al.*, 2004 ▶). The liquid sample was circulated by the magnetic gear pump at a flow rate of 180 ml min^−1^. The stability of the fluorescence signal and overlap of the X-ray and laser depend on the positional fluctuation of the liquid surface. We used a sapphire nozzle head to provide a stable liquid flow with a size of 7 mm (width) × 300 µm (thickness). The liquid surface was set at 45° against the X-ray.

A fast scintillation detector was located at 90° to the incident X-ray beam, coplanar with the X-ray polarization vector to avoid elastic scattering, and a Soller slit and a metal filter were set in front of the detector. The 794 kHz X-ray fluorescence signal was measured using a fast scintillation counter, which consists of a plastic scintillator (Saint-Gobain Crystals BC-420) with a nanosecond-order decay time, a 29% light output of NaI(Tl), and a photomultiplier tube (Hamamatsu H7195) with ϕ = 46 mm acceptance, 10 nA dark current and 3 × 10^6^ gain. The response time of the detector was fast enough to separate each X-ray pulse at 794 kHz. The pump and probe measurement was performed by detecting the fluorescence X-ray signals just after and just before the laser pulse using gated integrators (Stanford Instruments SR250) synchronized with the laser pulse (945 Hz). The output voltage from the gated integrators was converted to a frequency signal using a voltage-to-frequency (V–F) converter (Ohyo Koken Kogyo 733-1). The output pulses from the V–F converter were counted using a 100 MHz counter (Ortec 974) and further used to obtain the X-ray absorption spectrum.

**Figure 2:**
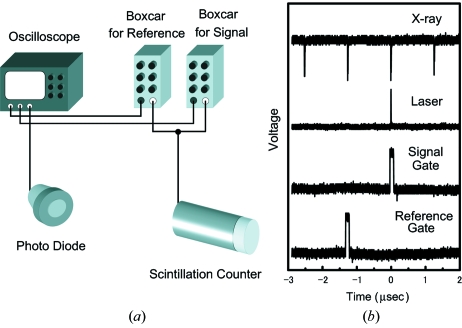
Schematic drawing of the detection system and the signal output observed on the oscilloscope during the time-resolved measurement.

Fig. 2(*a*) ▶ shows a schematic diagram of the measurement. The fluorescence X-ray signal from the scintillation counter was fed into the two inputs of the boxcar integrator as the signal and reference. The laser timing was sampled by a fast metal–semiconductor–metal photodiode (Hamamatsu G4176-03). All signals were monitored by a 2.5 GHz digital oscilloscope (Tektronix DPO 71254). Fig. 2(*b*) ▶ shows the signals measured by the oscilloscope during measurement.

### 2.3. X-ray positional feedback from the fixed-exit double-crystal monochromator and the figure-of-merit scan for the spatial overlap between the X-ray and laser

**Figure 3:**
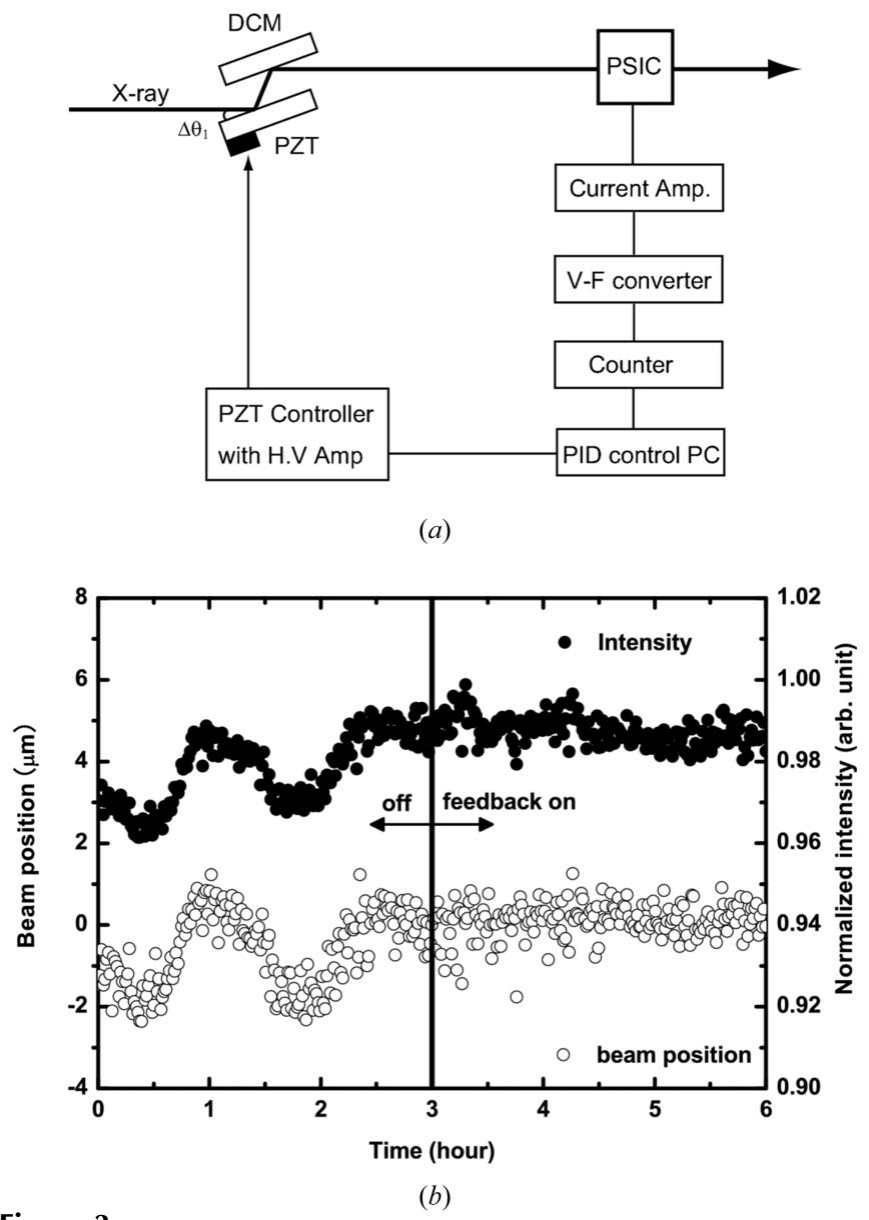
Stabilization of the beam position by the feedback system of the monochromator.

During the TR-XAS measurement it is important to produce a high-quality X-ray beam in order to measure a small differential signal between the photo-excited and ground states. This requisite involves accurate X-ray energy tuning (0.1 eV), a fixed beam position, and stable beam intensity over a wide range of energy scanning up to 1.5 keV. For this purpose the X-ray beam is generally monochromized by a fixed-exit double-crystal monochromator (DCM). The first crystal monochromatizes the incident white X-ray and the second crystal defines the X-ray energy. Because the incident white X-ray heats up the first crystal and causes thermal expansion of the crystal, the heat load causes instabilities in the energy, intensity and position of the exit beam from the DCM. We implemented a positional feedback control of the X-ray beam with the piezo actuator of Δθ_1_ to stabilize the beam position and intensity (Kudo & Tanida, 2007 ▶). A position-sensitive ion chamber (PSIC; Ohyo Koken Kogyo S-2403B) was set downstream of the DCM, and the output was converted to the positional signal. The beam position was stabilized by a proportional-integral-derivative (PID) control by monitoring the difference of the positional signal from the standard beam position. The feedback signal was converted to the voltage and led to the piezo actuator of the first crystal of the DCM. Fig. 3 ▶ shows the beam intensity measured by the *I*_0_ ionization chamber and the position measured using the PSIC at 7 keV with and without the feedback loop used for tuning Δθ_1_. Without the feedback loop the beam intensity and position are not stabilized, since the two crystals are not maintained in parallel. In contrast, a smooth and stable beam position and intensity are obtained with the feedback loop.

The spatial overlap of the X-ray and laser beam is typically achieved by positional scans using a slit or a pinhole. This method is not straightforward because two independent scans for laser and X-ray are required and the slit or pinhole must be aligned properly for each positional check. The most straightforward way to achieve this is by a laser positional scan using the X-ray signal itself as the figure of merit of the spatial overlap. This allows a reproducible alignment of the laser and X-ray during the experiment. This method can record the actual laser profile using a pump–probe signal at the actual laser intensity used in the experiment and makes it possible to estimate the laser shape and focal size without attenuating the laser power. In order to perform the figure-of-merit scan properly, the difference spectrum before and after photo-excitation must be known in advance. Thus, we align the X-ray and laser at the sample position by the pinhole scan first, and utilize the figure-of-merit scan as soon as the difference spectrum is successfully obtained.

**Figure 4:**
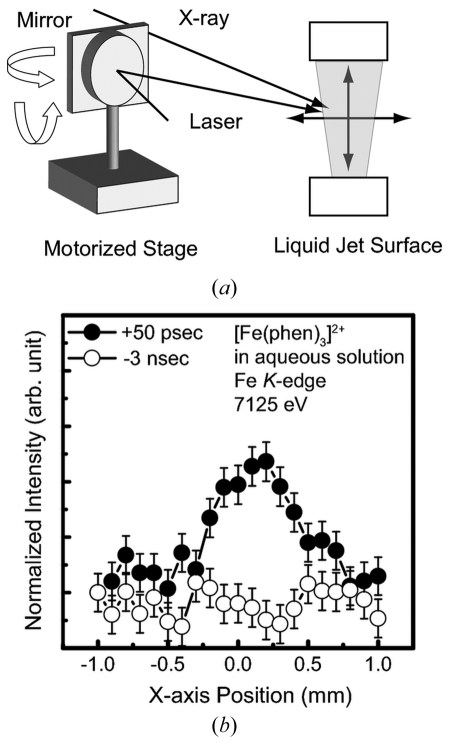
Figure-of-merit scan of the laser position by the motorized stage.

As shown in Fig. 4(*a*) ▶, a mirror just before the liquid jet is mounted on motorized rotational and swivel stages. First, the X-ray energy and laser delay timing are fixed at the maximum pump–probe signal, and the laser beam position is scanned using these two stages. The distance between the sample and the mirror was 300 mm, and a mirror rotational angle of 0.02° corresponds to 100 µm at the sample position. Fig. 4(*b*) ▶ shows the result of the figure-of-merit scan at 7125 eV; the overlap of both lights was achieved at 100 µm precision.

## 3. Pump–probe TR-XAS experiment of a spin crossover system

### 3.1. Spin crossover transition of [Fe^II^(phen)_3_]^2+^

Here we report a real example for the measurement of TR-XAS with 100 ps time resolution utilizing our experimental set-up. We have observed the photo-induced spin crossover (SCO) transition of [Fe^II^(phen)_3_]^2+^ in solution. It is known that this system undergoes structural change and electronic spin transition with a ∼680 ps decay time (McCusker *et al.*, 1993 ▶). This time scale of transition is suitable for our time resolution of 100 ps.

**Figure 5:**
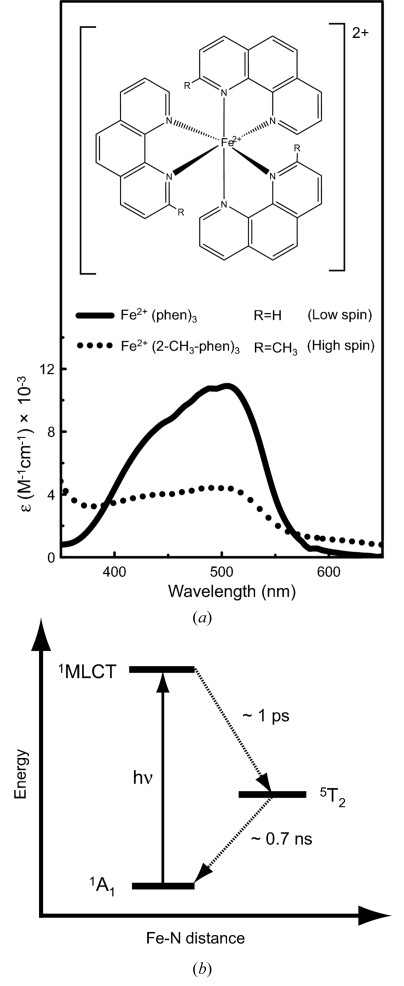
(*a*) Structure of [Fe^II^(phen)_3_]^2+^ and [Fe^II^(2-CH_3_-phen)_3_]^2+^ and their absorption spectra in the low-spin (solid line) and high-spin (dotted line) states. (*b*) Simplified energy diagram of the photo-induced spin crossover transition of [Fe^II^(phen)_3_]^2+^.

The structure of Fe^II^ complexes used in this study is shown in Fig. 5(*a*) ▶. [Fe^II^(phen)_3_]^2+^ is the six-coordinated low-spin complex in its ground state. The spectrum shown by the solid line is the absorption spectrum of [Fe^II^(phen)_3_]^2+^. The absorption peaks at 438, 477 and 510 nm correspond to the transition from ^1^A_1_ to ^1^MLCT (Jørgensen, 1957 ▶; Bosnich, 1968 ▶; Sullivan *et al.*, 1978 ▶). In addition to the [Fe^II^(phen)_3_]^2+^ complex, we prepared the high-spin analogue [Fe^II^(2-CH_3_-phen)_3_]^2+^ as a reference compound of the photo-excited state of [Fe^II^(phen)_3_]^2+^. The absorption spectrum of [Fe^II^(2-CH_3_-phen)_3_]^2+^, shown by the dotted line, is much weaker than that of the low-spin [Fe^II^(phen)_3_]^2+^. Fig. 5(*b*) ▶ shows an energy diagram of photo-induced SCO of [Fe^II^(phen)_3_]^2+^ suggested by the ultrafast transient absorption spectroscopy (McCusker *et al.*, 1993 ▶). The photo-excitation of the low-spin complex at the MLCT band induces a high-spin state ^5^T_2_, and the excited state decays from a high-spin to a low-spin state at a time constant of ∼680 ps. We observed this spin transition and structural change by TR-XAS at a 100 ps time resolution. [Fe^II^(phen)_3_]^2+^ and [Fe^II^(2-CH_3_-phen)_3_]^2+^ were prepared as reported previously (Pfeiffer & Christeleit, 1938 ▶; Irving *et al.*, 1953 ▶). All the reagents were reagent-grade and used without further purification.

### 3.2. Time-resolved XAS of [Fe^II^(phen)_3_]^2+^: results and discussion

**Figure 6:**
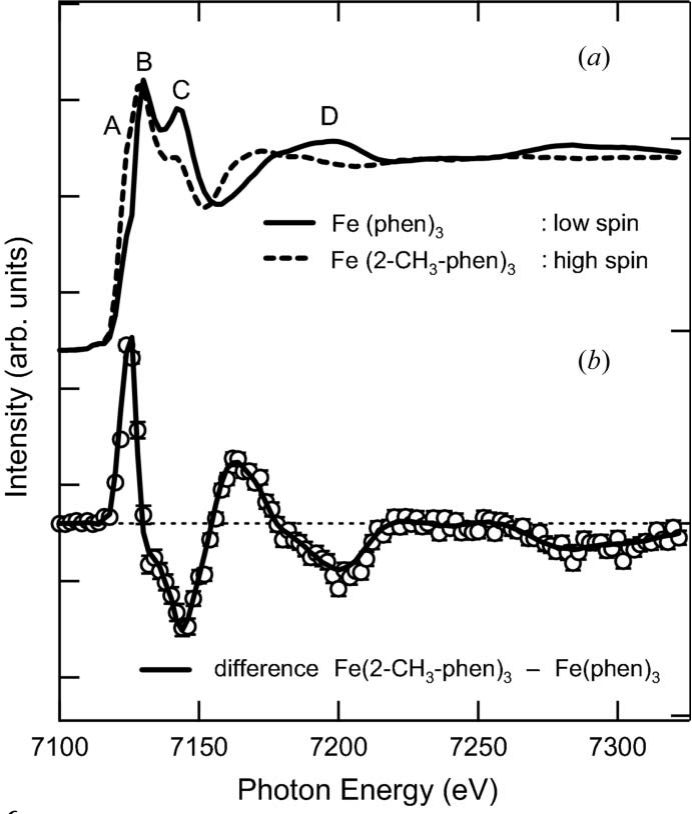
(*a*) Fe *K*-edge XANES spectra of a 50 m*M* aqueous solution of [Fe^II^(phen)_3_]^2+^ (solid line) and [Fe^II^(2-CH_3_-phen)_3_]^2+^ (dashed line). (*b*) Difference spectrum between [Fe^II^(phen)_3_]^2+^ and [Fe^II^(2-CH_3_-phen)_3_]^2+^ (solid line). Difference −1.3 µs before and 50 ps after excitation of [Fe^II^(phen)_3_]^2+^ (circles).

The upper part of Fig. 6 ▶ shows the steady-state XAS spectra of [Fe^II^(phen)_3_]^2+^ in the low-spin state and [Fe^II^(2-CH_3_-phen)_3_]^2+^ in the high-spin state in aqueous solution at the Fe *K*-edge. The difference spectrum of these steady-state spectra is shown in the lower part of Fig. 6 ▶ (solid line). The open circles show the transient difference spectrum of [Fe^II^(phen)_3_]^2+^ observed at 50 ps after laser excitation. The steady-state difference spectrum is scaled by 0.06, which accounts for the 6% photoexcitation yield of the ground-state species in solution by the laser irradiation. The spectral features labeled as *A* and *B* arise from the transition to the unoccupied Fe state. These unoccupied orbitals are caused by hybridization between the Fe (4*s*, 4*p*) and the N (2*p*) states. As the Fe—N bond length increases upon photo-excitation, going from low-spin to high-spin states, the overlap between the Fe and N orbitals diminishes. As a result, the ligand field strength decreases and a hole on the Fe 4*p* orbital is generated, leading to the enhancement of the dipole transition from 1*s* to 4*p*, as shown by spectral feature *A*. Since this transition involves a contribution of the orbital that is formally anti-bonding, the stretching of the bond length lowers the energy of the un­occupied Fe—N state, accounting for the red shift of feature *B*. Features *C* and *D* are attributed to multiple-scattering processes of the photoelectron (Hannay *et al.*, 1997 ▶; Briois *et al.*, 2001 ▶; Boillot *et al.*, 2002 ▶).

**Figure 7:**
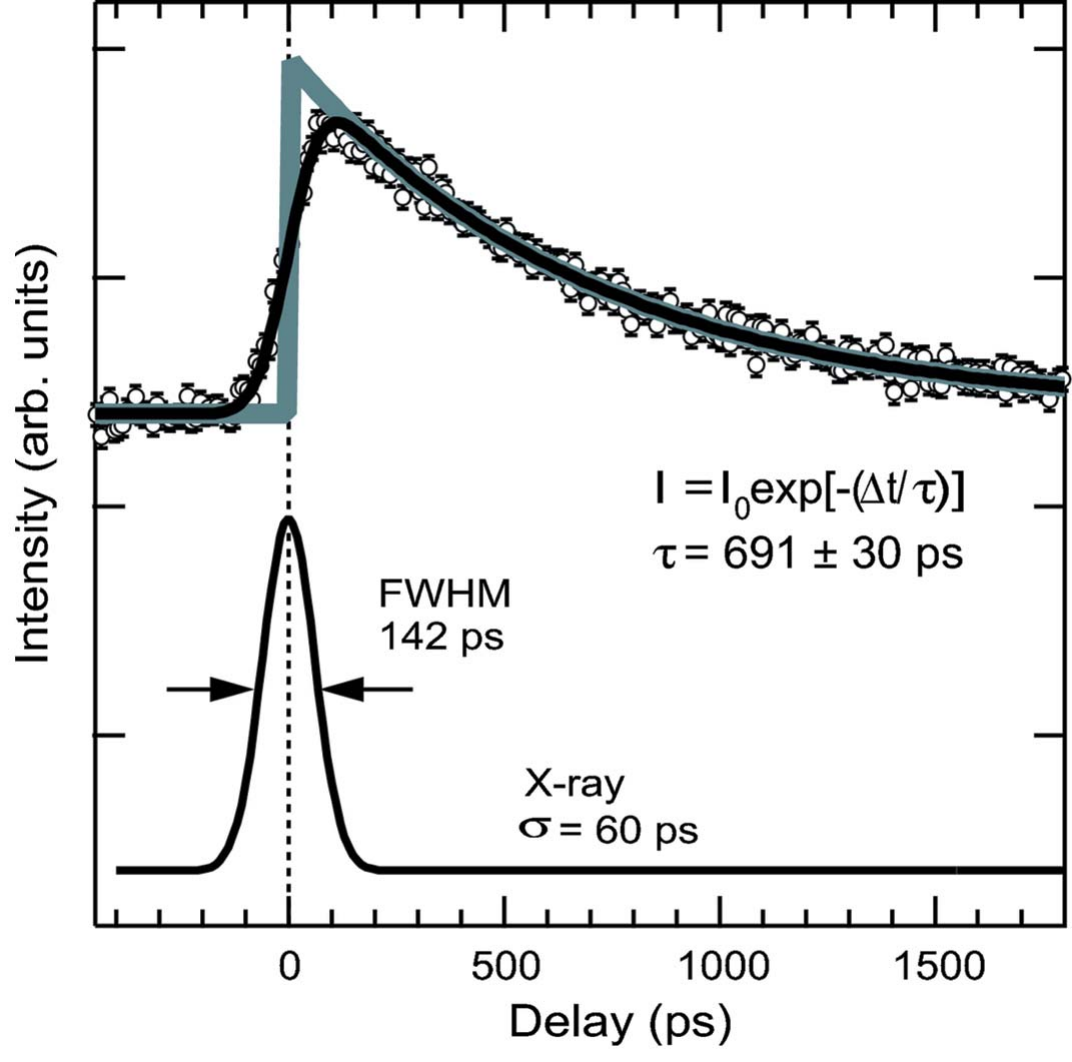
Time course of the difference signal at 7125 eV. Fit of the data using a single exponential function convoluted with the instrument function (σ = 60 ps) (solid line).

As discussed previously, spectral feature *A* directly reflects the change in the bond length between Fe and N atoms. Therefore the observed intensity enhancement at this photon energy can be utilized as a proper probe of the photo-induced structural change with the spin state transition. In order to study the time evolution of the spectral features, we measured the time course of feature *A* at 7125 eV, as shown in Fig. 7 ▶. The temporal evolution of the difference X-ray signal is modeled as a single exponential function convoluted with the instrument response function. The instrument function is estimated by the pulse duration of the X-ray and modeled by a Gaussian error function (thick solid curve) with σ ≃ 60 ps. This result indicates that the structural evolution after the ^1^A_1_ to ^1^MLCT excitation of the low-spin compound is complete within our time resolution. The decay time of 700 ps agrees well with the life-time estimated by ultra-fast spectroscopy (McCusker *et al.*, 1993 ▶). The structural changes will be obtained by further analysis of the TR-EXAFS, which is directly linked to the transiently generated high-spin species of the [Fe^II^(phen)_3_]^2+^ compound in solution. A detailed structural analysis of the TR-XAS is now underway, and the results of the analysis will be presented elsewhere.

## 4. Concluding remarks

We have presented an experimental set-up for time-resolved X-ray absorption spectroscopy with 100 ps time resolution at beamline NW14A at the PF-AR. We have demonstrated that the X-ray positional feedback of the monochromator and the figure-of-merit scan of the laser beam position are powerful tools for achieving the efficient observation of precise and detailed photo-induced change in TR-XAS. By utilizing this feedback and scanning system, a photo-induced transient signal of a spin crossover reaction of the [Fe^II^(phen)_3_]^2+^ complex in water was successfully detected. Further analysis of the pre-edge region and EXAFS structure will reveal the details of the photo-induced spin state and structure.
